# *Bifidobacterium longum* subsp. *infantis* CCFM1426 enhances the anti-colitic effect of vitamin A via retinoic acid restoration and gut microbiota modulation in ulcerative colitis mice

**DOI:** 10.3389/fnut.2025.1644649

**Published:** 2025-07-24

**Authors:** Xihua Yu, Liming Huang, Yi Wang, Liuruolan Li, Wenwei Lu, Zhijian Zhang, Hongchao Wang

**Affiliations:** ^1^Sinopharm Xingsha Pharmaceutical (Xiamen) Co., Ltd., Xiamen, Fujian, China; ^2^Xiamen Key Laboratory of Maternal and Infant Health and Nutrition Products, Xiamen, Fujian, China; ^3^State Key Laboratory of Food Science and Resources, Jiangnan University, Wuxi, Jiangsu, China; ^4^School of Food Science and Technology, Jiangnan University, Wuxi, Jiangsu, China; ^5^Department of Nephrology, The Affiliated Wuxi People’s Hospital of Nanjing Medical University, Wuxi People’s Hospital, Wuxi Medical Center, Nanjing Medical University, Wuxi, Jiangsu, China

**Keywords:** ulcerative colitis, vitamin A, retinoic acid, *Bifidobacterium longum* subsp. *infantis*, gut microbiota

## Abstract

**Background:**

Ulcerative colitis (UC) is a chronic inflammatory bowel disease with increasing global prevalence, making it a significant health concern. Although vitamin A (VA) plays a beneficial role in UC management, its therapeutic efficacy is limited by impaired absorption and disrupted retinoic acid (RA) metabolism. Gut microbiota are known to influence VA metabolic pathways, offering potential targets to enhance VA bioavailability and efficacy.

**Methods:**

A dextran sulphate sodium (DSS)-induced mouse model of colitis was established to evaluate the therapeutic effects of co-administering *Bifidobacterium longum* subsp. *infantis* CCFM1426 with vitamin A. Body weight, disease activity index (DAI) and colon length were monitored in mice with DSS-induced colitis. Serum levels of intestinal injury markers, inflammatory cytokines, antioxidant enzymes and colonic RA levels were measured using ELISA kits. Metagenomic analysis investigated gut microbiota composition.

**Results:**

It was indicated that the VA and CCFM1426 combination significantly improved colon length and DAI, enhanced serum levels of intestinal injury markers (lipopolysaccharide-binding protein, intestinal fatty acid-binding protein, diamine oxidase) and cytokines (IL-6, TNF-*α*, IL-10), and restored antioxidant capacity. The combination demonstrated superior efficacy in colonic RA levels and contributed to gut microbiota diversity restoration. Metabolomics analysis showed that colitis mice treated with the combination had higher levels of eicosapentaenoic acid, adenosine and anandamide.

**Conclusion:**

These findings provide novel evidence that co-administration of CCFM1426 and VA synergistically alleviates colitis by enhancing RA bioavailability through microbiota-dependent pathways.

## 1 Introduction

Inflammatory bowel disease (IBD) is a chronic condition characterised by gastrointestinal tract inflammation (1). The rising global prevalence of IBD presents significant challenges to public health and safety (2, 3). Ulcerative colitis (UC), a common type of IBD, primarily affects the colon and rectum, compromising the mucosal barrier and colonic intestinal epithelium, leading to intestinal barrier dysfunction (4). Clinical symptoms of UC include diarrhoea, bloody stools and abdominal pain, often associated with dysregulated intestinal immune systems, barrier damage and imbalanced intestinal flora homeostasis (5). At present, the clinical treatment of UC mainly relies on medications such as 5-aminosalicylic acid (5-ASA), corticosteroids, and immunomodulators (6). However, these treatment approaches are associated with issues such as drug dependence and resistance, and long-term use may even lead to serious adverse effects (7). Therefore, identifying safe and effective strategies for UC management is of critical importance.

Currently, UC treatment strategies targeting nutrition and the gut microbiota are gaining increasing attention (8, 9). Vitamin A (VA) is an essential micronutrient for growth and development with numerous physiological functions including maintenance of mucosal integrity, preservation of visual functions, enhancement of immune response and participation in cell growth and differentiation (10). Supplementation with VA has been shown to mitigate UC by restoring microbial diversity in the gut of colitis mice and alleviating intestinal damage and inflammation *in vivo* (11). In individuals with UC, VA treatment can effectively decrease the disease activity index (DAI) (12). Retinoic acid (RA), one of VA’s primary active forms, is involved in crucial physiological processes (13). RA plays a vital role during embryonic and postnatal periods and is recognised as a substantial regulator of mammalian gene expression (14). Notably, RA has been shown to contribute to the maintenance of intestinal barrier function, the regulation of mucosal immune responses, and the modulation of host-microbiota interactions, thereby supporting intestinal homeostasis (15). Although VA has been shown to be beneficial in inflammatory conditions, mucosal damage in UC patients often results in decreased serum VA levels and impaired intestinal absorption (16–18). Furthermore, dysregulated RA biosynthesis in UC is further exacerbated by changes in the expression of the enzymes involved in VA metabolism, such as decreased ALDH1A1 and increased CYP26A1, which worsen inflammation and barrier dysfunction (19).

Recent studies have uncovered a critical role for gut microbiota in modulating host vitamin A metabolism. Intestinal flora exhibit host-independent metabolism of VA, producing retinaldehyde, RA and other active substances (20). These microbiota-derived retinoids can modulate the expression of serum amyloid A (SAA) and retinaldehyde dehydrogenases (ALDH) in intestinal epithelial cells, thereby regulating host RA levels, modulating intestinal immunity, and alleviating gut inflammation (21, 22). The impairment of RA-mediated signal transduction in the intestine and systemic induction in UC may be associated with disturbed VA metabolism in gut microbiota due to ecological dysregulation (16, 17). Therefore, modulating gut microbiota to regulate host VA metabolism may represent an effective strategy for alleviating colitis.

Supplementing with probiotics is an effective way to regulate the gut microbiota (23). *Bifidobacterium longum* subsp. *infantis* is a well-documented probiotic with anti-inflammatory properties and proven benefits in maintaining gut barrier integrity. The strain FJSYZ1M3 of *B. infantis* maintains the integrity of the intestinal barrier and produces beneficial metabolites such as butyrate (24). The combination of *B. infantis* and xylo-oligosaccharides has been shown to enhance colonic epithelial barrier function more effectively than either the probiotic or the prebiotic alone (25). In addition, probiotic supplementation in early infancy can enhance the protective effects of prenatal vitamin supplementation (26). The combination of lactic acid bacteria and vitamin E metabolites can exert a synergistic effect in alleviating colitis (27). Therefore, We propose that co-supplementation with VA and *B. infantis* may synergistically alleviate UC by altering the gut microbiota and promoting RA bioavailability.

Accordingly, this study explored the therapeutic effects of combined supplementation with *B. infantis* CCFM1426 and VA in a dextran sulphate sodium (DSS)-induced colitis mouse model. We hypothesised that this combination would synergistically enhance colonic RA levels, modulate gut microbiota composition, and ultimately improve colitis outcomes beyond VA supplementation alone. Furthermore, we investigated underlying mechanisms involving intestinal barrier markers, inflammatory cytokines, antioxidant enzymes, microbial shifts, and faecal metabolites to elucidate the multi-dimensional benefits of this nutrition–microbiota synergistic approach.

## 2 Materials and methods

### 2.1 Bacterial strains

The bacterial strain CCFM1426 used in this study was obtained from the Culture Collections of Food Microbiology (CCFM) at Jiangnan University, Wuxi, China. Following three successive subcultures in MRS broth, bacteria were harvested by centrifugation (8,000 rpm, 15 min, 4°C), resuspended in 30% glycerol, and stored at −80°C. The final bacterial suspension for oral gavage was standardised to approximately 109 CFU/mL.

### 2.2 Preparation of vitamin A and retinoic acid for oral gavage

VA was purchased from J&K Scientific Ltd. (Beijing, China), whilst RA was obtained from Titan Scientific Co., Ltd. (Shanghai, China). For each preparation, 0.5 mg of VA or RA was dissolved in 200 μL of plant oil and thoroughly vortexed until completely dissolved (28, 29). The resulting solutions were freshly prepared before administration and protected from light throughout the preparation process.

### 2.3 Experimental design

Six-week-old male C57BL/6 J mice were acquired from Vital River Laboratory Animal Technology Co., Ltd. (Jiaxing, China) and maintained in specific pathogen-free (SPF) conditions. The laboratory environment was maintained at 20–26°C with 40–70% relative humidity and a 12-h light/dark cycle. All mice received ad libitum access to food and water. The experimental protocols were approved by the Ethics Committee of Experimental Animals of Jiangnan University (JN. No20220915b1281110[318]).

Following a one-week acclimatisation period, 30 mice were randomly allocated into five groups (*n* = 6 per group): control, model, VA, RA, and CCFM1426 + VA. The UC model was established using previously described methods (30). Briefly, mice in the model group received drinking water supplemented with 3% (w/v) dextran sodium sulphate (molecular weight, 36–50 kDa) for 7 days, followed by administration of 200 μL 0.9% saline for 6 days. The intervention doses of VA and RA were determined according to previously published methods (28, 29). The VA and RA groups received 200 μL of vegetable oil solution containing 0.5 mg of VA or RA, respectively, for six consecutive days. The CCFM1426 + VA group received 10^9^ CFU/mL of CCFM1426 bacterial suspension combined with 0.5 mg VA in 200 μL of vegetable oil solution for 6 days.

### 2.4 Weight measurement and evaluation of colitis mice

Body weight was measured daily prior to gavage. The percentage of body weight change (%) was calculated as:

$$\begin{array}{l} Percentageofbodyweight\;\left(\%\right)=\left(currentweight\right)\\ \;\;\;\;\;\;\;\;\;\;\;\;\;\;\;\;\;\;\;\;\;\;\;\;\;\;\;\;\;\;\;\;\;\;\;\;\;\;\;\;\;\;\;\;\;\;\;\;\;\;\;\;\;\;\;\;\;\;\;\;\;\;\;\;\;\;\;\;\;\;\;\;\;/\left(8th\;day\;weight\right)\times 100\% \end{array}$$

DAI was assessed according to previously established methods, based on changes in body weight, presence of blood in stool, and faecal consistency. Occult blood in stool was detected using an OB (occult blood) reagent kit (Cell Science & Technology Institute, China). The DAI score was calculated based on the criteria listed in Table 1.

**Table 1:** Scoring criteria for calculating DAI.

Score	Weight loss (%)	Stool consistency	Blood in stools
0	1–5	Normal	No colour change upon addition of the reagent
1	5–10	Soft but well formed	Purplish-red coloration appears within 1–2 min
2	10–15	Soft but not formed	Purplish-red coloration appears within 1 min
3	15–20	Slight diarrhoea	Purplish-red coloration appears within 10 s
4	More than 20	Watery diarrhoea	Immediate purplish-red coloration upon reagent addition

### 2.5 Histopathological changes in mouse colon tissues

Colon tissues were excised, fixed in 4% paraformaldehyde for a minimum of 24 h, and subsequently embedded in paraffin. Tissue sections were cut and mounted with neutral gum. Haematoxylin and eosin (H&E) staining was performed, and images were captured using a microscopy imaging system.

### 2.6 Detection of serum biomarkers for intestinal barrier damage

On day 14 of the experiment, mice were sacrificed and blood samples were collected via orbital extraction. The blood was collected in sterile centrifuge tubes and centrifuged at 3000 rpm at 4°C for 15 min. The serum was then transferred to sterile PCR tubes, with remaining aliquots stored at −80°C for subsequent analyses. Serum levels of intestinal fatty acid-binding protein (I-FABP), lipopolysaccharide-binding protein (LBP), and diamine oxidase (DAO) were quantified using enzyme-linked immunosorbent assay (ELISA) kits (Enzyme-linked Biotechnology Co., Ltd.).

### 2.7 Measurement of RA, inflammatory cytokines, and antioxidant enzyme levels in the colon

Collected colon tissues were homogenised using a tissue grinder, followed by centrifugation to obtain the supernatant. Protein content in the supernatant was quantified using the BCA Protein Assay Kit (Beyotime). Enzyme-linked immunosorbent assay (ELISA) kits (Enzyme-linked Biotechnology Co., Ltd., Shanghai, China) were employed to assess levels of retinoic acid, glutathione peroxidase (GSH-PX), catalase (CAT), and superoxide dismutase (SOD) activity in colon tissues. Additionally, inflammatory cytokines, including tumour necrosis factor-alpha (TNF-*α*), interleukin-1 beta (IL-1β), interleukin-6 (IL-6), and interleukin-10 (IL-10), were measured in colon tissues using the same ELISA kits, following the manufacturer’s instructions.

### 2.8 Untargeted metabolomics analysis of faeces

Faecal sample pre-treatment was conducted according to previously established protocols. Briefly, non-targeted metabolite analysis was performed using a Vanquish UHPLC Q-Exactive Plus MS system with a Phenomenex UHPLC Kinetex C18 column (2.6 μm, 2.1 × 100 mm). The analysis employed a binary solvent system comprising mobile phase A (0.01% acetic acid in H2O) and mobile phase B (50% ACN + 50% IPA). The gradient elution profile was programmed as follows: 0–1 min, 1% B; 1–8 min, 1 to 99% B; 8–9 min, 99% B; 9–9.1 min, 99 to 1% B; 9.1–12 min, 1% B. The column was maintained at 35°C with a flow rate of 300 μL/min and an injection volume of 2 μL. Metabolites with an mzCloud Best Match score > 85 were considered confidently identified. The processed metabolomic data were analyzed using MetaboAnalyst 6.0.^1^ Prior to statistical analysis, metabolites with a relative standard deviation (RSD) >20% were excluded to ensure data quality (31). The remaining data were subjected to log10 transformation and autoscaling to normalise distributions and reduce heteroscedasticity.

### 2.9 Metagenomic analysis of mouse faeces

Metagenomic analysis of mouse faecal samples (*n* = 5 for CON and MOD; *n* = 4 for treatment groups) was performed by Novogene Co. Ltd. (Beijing, China). In brief, low-quality sequences from raw data generated by the DNBSEQ-T7 platform were filtered using Trimmomatic (version 0.39). Sequences with average base quality scores below 30 were trimmed, whilst those exceeding 60 bp were retained for subsequent analysis. Host genomic sequences were removed using BWA (version 0.7.17), Samtools (version 1.17), and BEDTools (version 2.30.0), and the cleaned sequences were aligned to the Genome Reference Consortium Mouse Build 39 (GRCm39). Intestinal microbiota species were annotated using MetaPhlAn4. Spearman correlation heatmaps were generated using an online tool.^2^ Beta diversity and linear discriminant analysis of effect size (LEfSe) were conducted using https://www.bioincloud.tech/. The metagenomic sequencing data generated have been deposited in the NCBI under the BioProject accession number PRJNA1275596.

### 2.10 Statistical analysis

Data analysis and visualisation were performed using GraphPad Prism (version 9.5.0) and OriginPro 2022. Results are presented as mean ± standard error of the mean (SEM). Group differences were evaluated using one-way analysis of variance (ANOVA) followed by Duncan’s multiple range test. Significant inter-group differences were analysed using GraphPad Prism (version 9.5.0). Unless otherwise specified, results indicate differential comparisons with the model group; “*” indicates *p* < 0.05, “**” indicates *p* < 0.01, “***” indicates *p* < 0.001, “****” indicates *p* < 0.0001.

## 3 Results

### 3.1 VA and CCFM1426 combination alleviates changes in body weight and colonic tissues in colitis mice

**Figure 1:**
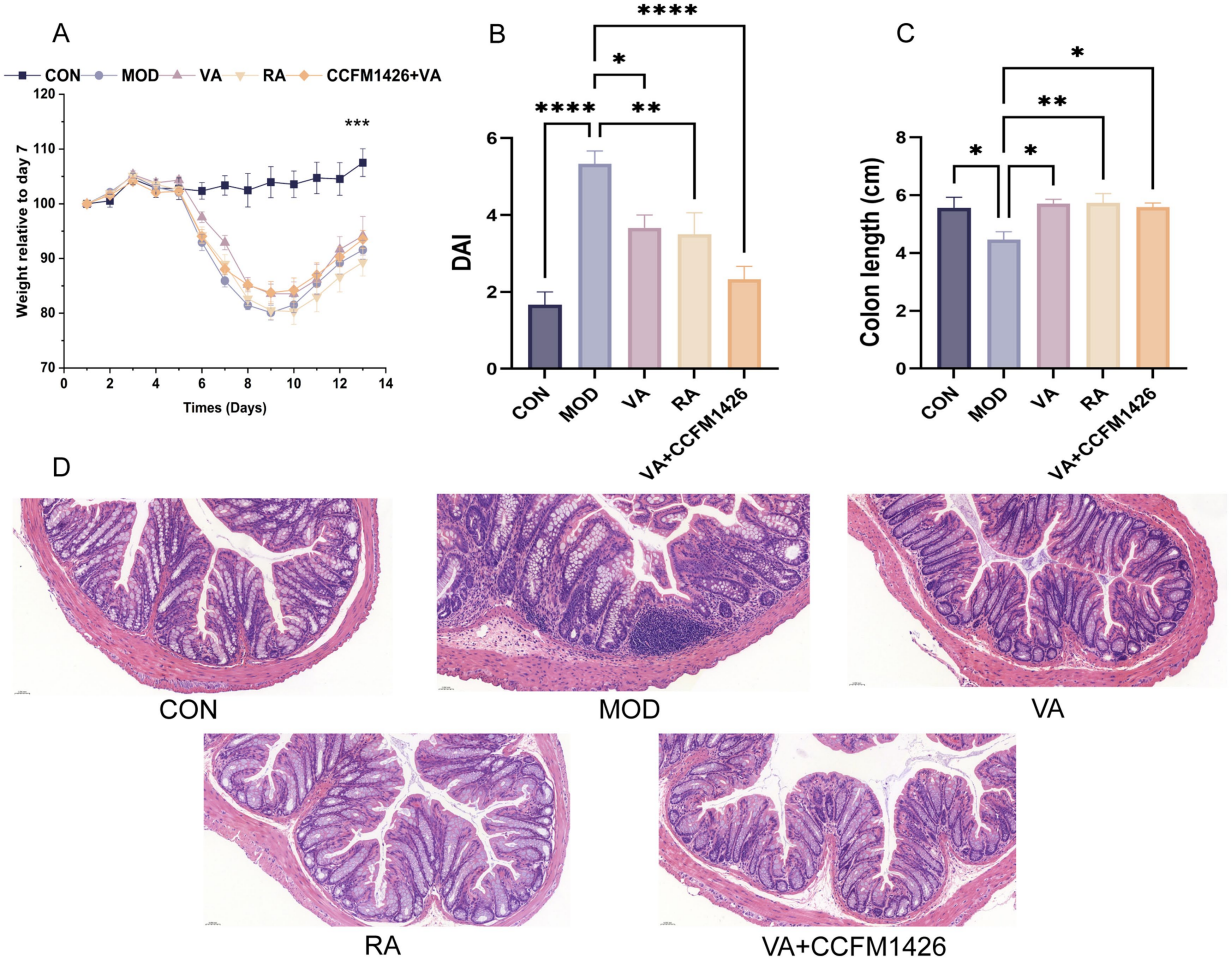
Vitamin A and CCFM1426 combination alleviates changes in body weight and colonic tissues in colitis mice. **(A)** Body weight. **(B)** DAI scores. **(C)** Colon length. **(D)** Representative images of H&E-stained colon sections, ×20.

Body weight decreased in all treatment groups compared to the control group, although the VA and CCFM1426 combination did not significantly affect body weight relative to the model group (Figure 1A). DAI was significantly elevated in the model group compared to the control group (Figure 1B; *p* < 0.0001). Treatment with the VA and CCFM1426 combination significantly reduced DAI in colitis mice (*p* < 0.0001). As depicted in Figure 1C, colon length was significantly shorter in the model group than in the control group (*p* < 0.001). Supplementation with VA, RA, or the combination significantly increased colon length in colitis mice (*p* < 0.05). Histological examination (Figure 1D) revealed that the model group exhibited disrupted colonic crypts, upward displacement, crypt distortion, inflammatory cell infiltration, and increased basal plasma cells with haemorrhage. Both RA and the combination treatment reduced inflammation, decreased cell infiltration, restored crypt structure, and improved crypt morphology without significant bleeding.

### 3.2 The combination of CCFM1426 and VA reduces serum biomarkers of intestinal barrier damage in colitis mice

**Figure 2:**
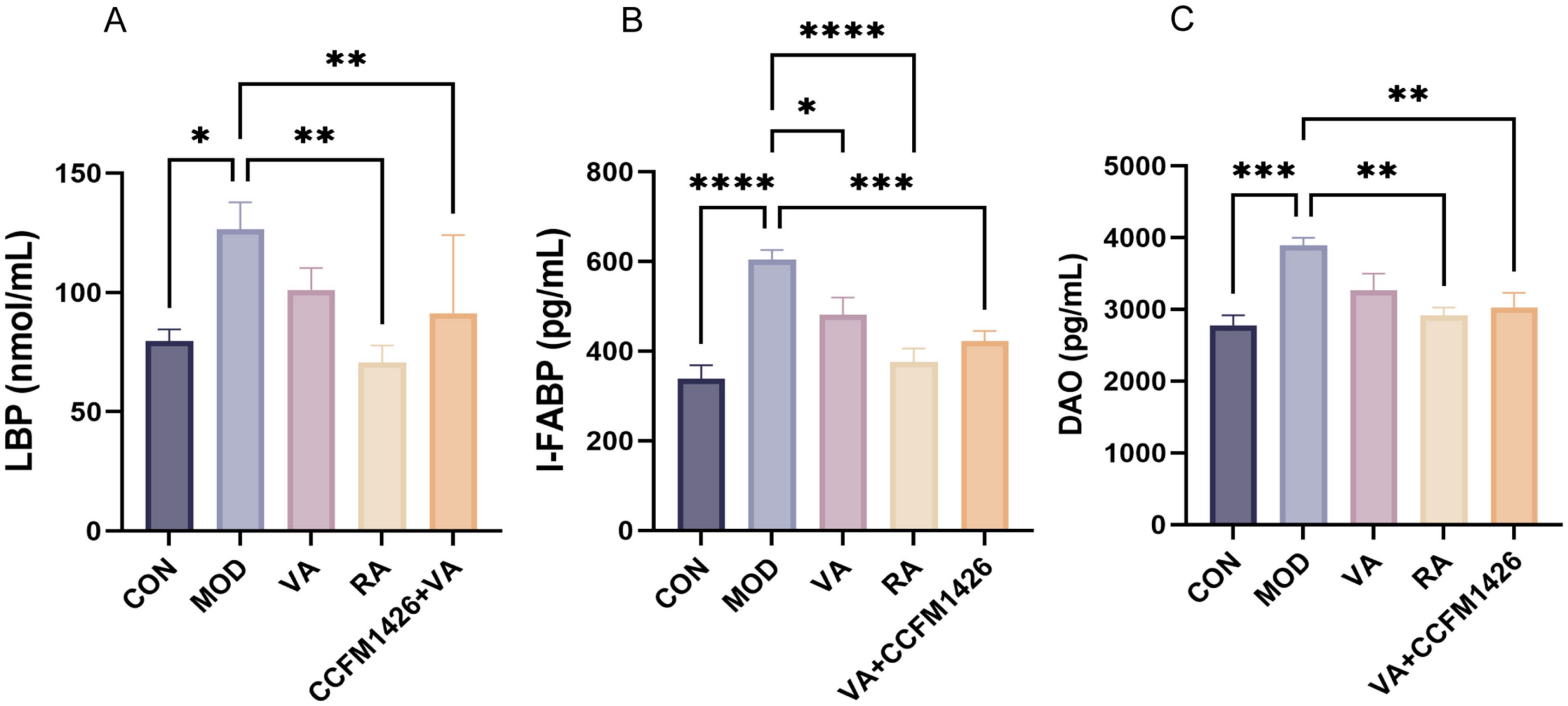
The combination of CCFM1426 and vitamin A reduces serum biomarkers of intestinal barrier damage in colitis mice. **(A)** LBP. **(B)** I-FABP. **(C)** DAO.

To investigate the effects of probiotic treatment on intestinal barrier function, serum levels of DAO, LBP, and I-FABP were measured. As shown in Figures 2A–C, the model group exhibited significantly higher serum levels of these markers compared to the control group (*p* < 0.05). In colitis mice, serum levels of DAO, LBP, and I-FABP were reduced following administration of VA, RA, or the combination. However, VA treatment alone did not significantly reduce LBP and DAO levels, whereas RA treatment significantly decreased all three markers (*p* < 0.01; Figures 2A–C). The CCFM1426 and VA combination significantly reduced all three marker levels, demonstrating superior therapeutic efficacy compared to VA alone (*p* < 0.01; Figures 2A–C).

### 3.3 The combination of CCFM1426 and VA modulates RA and inflammatory cytokine levels in colitis mice

**Figure 3:**
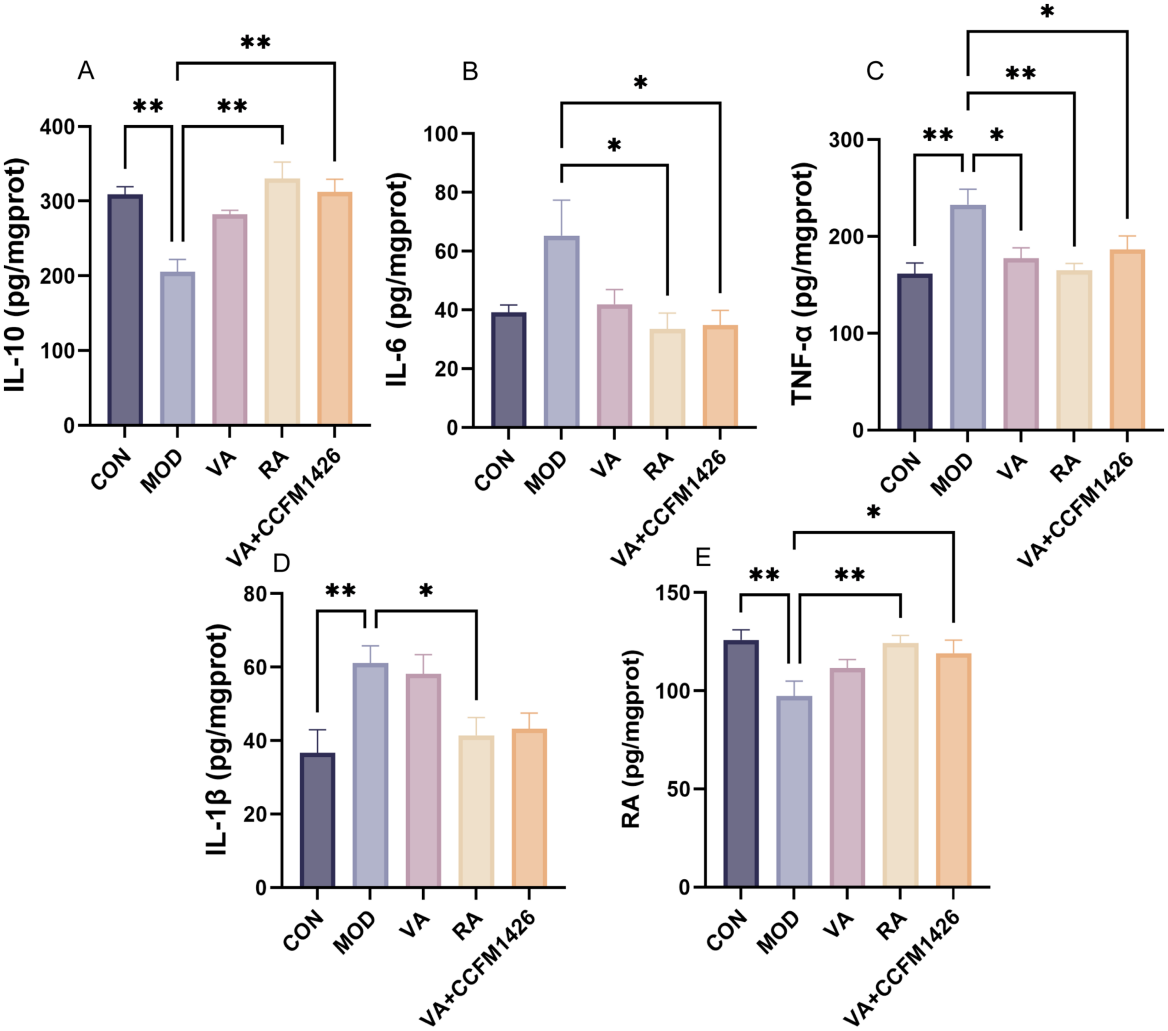
The combination of CCFM1426 and Vitamin A modulates RA and inflammatory cytokine levels in colitis mice. **(A)** IL-10. **(B)** IL-6. **(C)** TNF-*α*. **(D)** IL-1β. **(E)** RA.

The anti-inflammatory effects of VA, RA, and their combination in DSS-treated mice were assessed by measuring pro-inflammatory cytokines (TNF-*α*, IL-6, and IL-1β) and the anti-inflammatory cytokine IL-10. The model group exhibited decreased IL-10 levels compared to the control group (Figure 3A). Furthermore, the model group demonstrated significantly elevated levels of TNF-α, IL-1β, and IL-6 in colonic tissues compared to the control group (*p* < 0.01; Figures 3B–D). Treatment with VA, RA, or the combination modulated both pro- and anti-inflammatory cytokine profiles. In colitis mice, the CCFM1426 and VA combination significantly reduced IL-6 and TNF-α levels whilst increasing IL-10 levels (*p* < 0.05; Figures 3A–C). Similarly, RA treatment significantly reduced TNF-α, IL-1β, and IL-6 levels whilst markedly increasing IL-10 levels in the model group (*p* < 0.05; Figures 3A–D). Additionally, DSS-treated mice exhibited substantially lower colonic RA levels compared to control mice (*p* < 0.001; Figure 3E). Treatment with RA or the combination significantly improved colonic RA levels (*p* < 0.05), with the combination proving more effective than VA alone, although the difference was not statistically significant. These findings suggest that CCFM1426 enhances the VA metabolic capacity of the gut microbiota in colitis mice, thereby boosting gut-derived RA production. This RA subsequently enters intestinal tissues through the epithelium, elevating colonic RA levels, which aligns with previous research findings (21).

### 3.4 The combination of VA and CCFM1426 improves antioxidant enzyme levels in the colon of mice with colitis

**Figure 4:**
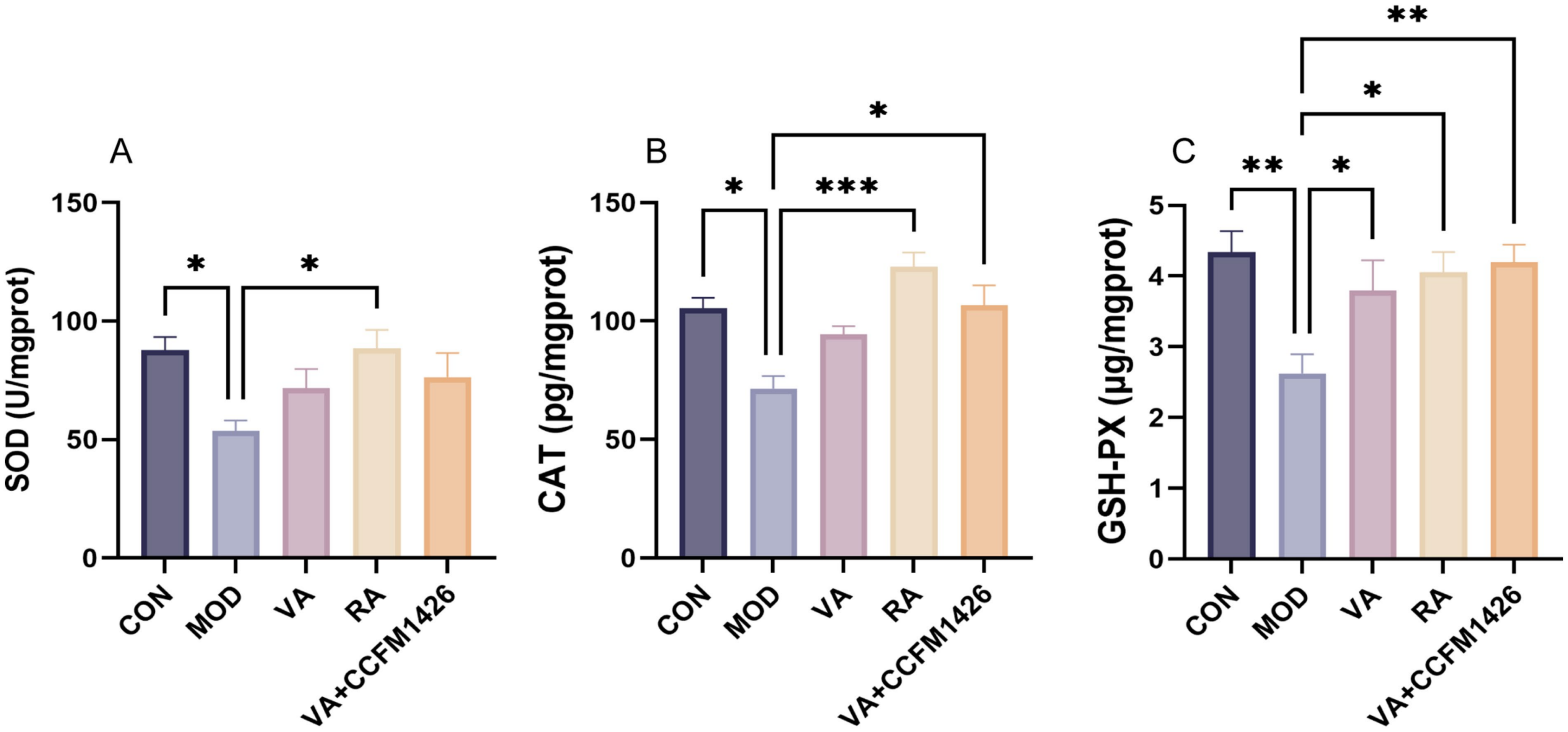
The combination of VA and CCFM1426 improves antioxidant enzyme levels in the colon of mice with colitis. **(A)** SOD. **(B)** CAT. **(C)** GSH-PX.

The levels of antioxidant enzymes (CAT, GSH-PX, and SOD) were significantly lower in the colon tissues of the model group compared to the control group (*p* < 0.05; Figures 4A–C). Following treatment with RA, the activity of these enzymes was notably increased compared to the model group (*p* < 0.05; Figures 4A–C). Specifically, the concentrations of CAT and GSH-PX were significantly higher in the CCFM1426 and VA combination group compared to the model group (*p* < 0.05; Figures 4A,C). The combination exhibited superior antioxidant capabilities compared to VA alone.

### 3.5 The combination of vitamin A and CCFM1426 improves colitis by modulating the gut microbiota composition

**Figure 5:**
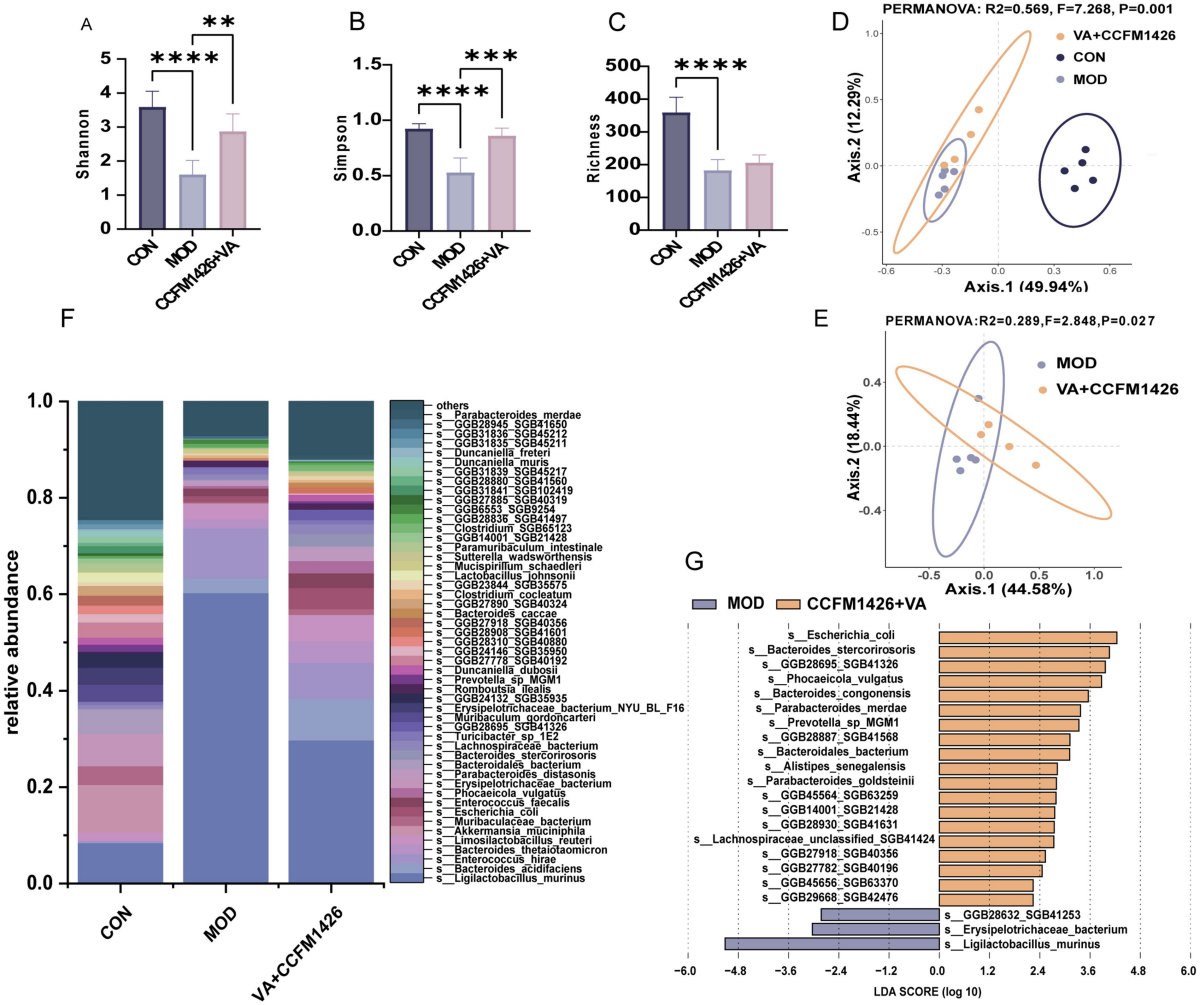
The combination of vitamin A and CCFM1426 improves colitis by modulating the gut microbiota composition. **(A)** Shannon. **(B)** Simpson. **(C)** Richness. **(D)** Bray-Curtis Principal Coordinate Analysis (PCoA) of all groups. **(E)** PCoA analysis of the CCFM1426 and Vitamin A combination group compared to the model group. **(F)** Relative abundance of the gut microbiota at the species level. **(G)** Results of linear discriminant analysis effect size (LEfSe).

Various *α*-diversity indices, including Shannon index, Simpson index, and richness, were calculated. As shown in Figures 5A–C, the α-diversity indices of mice in the control group were significantly higher than those in the model group (*p* < 0.0001). Treatment with the combination of CCFM1426 and VA significantly improved the Shannon and Simpson indices of mice with colitis compared to the model group, whilst the richness was not statistically significant (Figures 5A–C). The *β*-diversity results (Figures 5D–E) indicated that the intestinal flora of the model group exhibited significant differences compared to both the control group and the combination of CCFM1426 and VA group (*R*^2^ = 0.289, *p* = 0.027). Thus, DSS-induced colitis disrupted homeostasis of the intestinal flora in mice (*p* < 0.05). At the species level, the combination induced changes in the gut microbiota composition (Figure 5F). As shown in Figure 5G, LDA score results indicated higher relative abundances of *Ligilactobacillus_murinus* and *Erysipelotrichaceae*_bacterium in the model group compared to the combination groups. The administration of the combination of CCFM1426 and VA increased the abundance of *Escherichia_coli*, *Bacteroides_stercorirosoris*, *Phocaeicola_vulgatus*, *Bacteroides_congonensis*, and *Parabacteroides_merdae*.

### 3.6 The combination of vitamin A and CCFM1426 improves colitis by modulating the faecal metabolic phenotype

**Figure 6:**
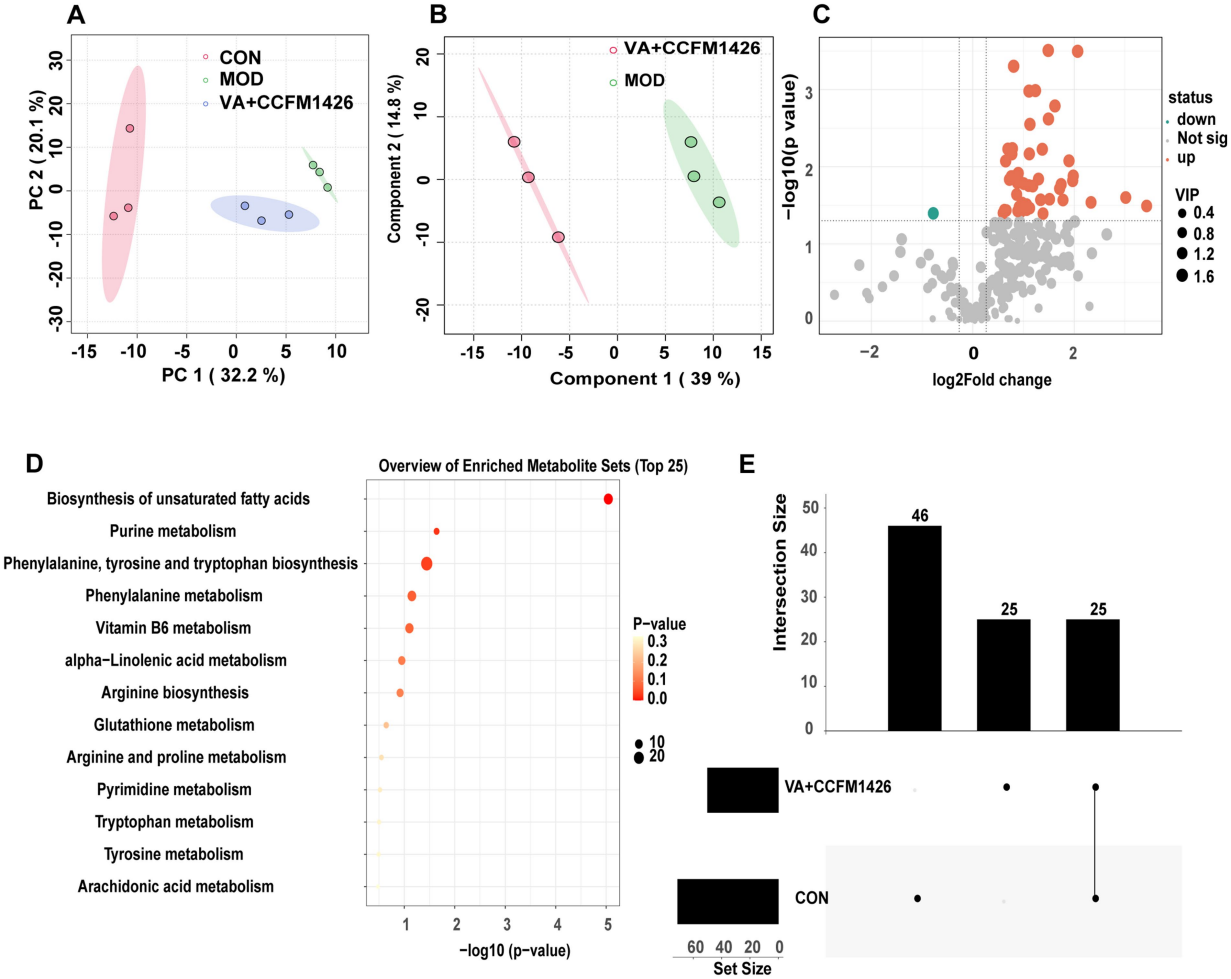
The combination of vitamin A and CCFM1426 improves colitis by modulating the faecal metabolic phenotype. **(A)** Principal component analysis (PCA) of all groups. **(B)** Partial least squares discrimination analysis of the CCFM1426 and Vitamin A combination group compared to the model group. **(C)** Volcano maps of differentially abundant metabolites of the CCFM1426 and Vitamin A combination group compared to the model group. **(D)** KEGG pathway enrichment analysis of the CCFM1426 and Vitamin A combination group compared to the model group. **(E)** UpSet plot of differential metabolites amongst groups.

Changes in faecal metabolites following treatment with the combination of VA and CCFM1426 were further evaluated in mice using liquid chromatography-mass spectrometry (LC–MS). Principal component analysis (PCA) and partial least squares discriminant analysis (PLS-DA) revealed that the faecal metabolite composition in the group receiving the combined intervention of vitamin A and CCFM1426 was different from that of the model group (Figures 6A,B). As described in previous studies, volcano plot analysis, applying thresholds of fold change (FC) > 1.2 or < 0.83, and a *p*-value <0.05, revealed 51 metabolites that were differentially abundant between the MOD group and the VA + CCFM1426 intervention group (Figure 6C) (31). KEGG pathway enrichment analysis indicated that the differentially abundant metabolites were mainly associated with several key metabolic pathways, including purine metabolism, biosynthesis of unsaturated fatty acids, as well as phenylalanine, tyrosine and tryptophan biosynthesis (Figure 6D). Specifically, guanine, adenosine, and urate were markedly enriched in the purine metabolism pathway. The significant variation in phenylalanine levels was closely associated with phenylalanine, tyrosine and tryptophan biosynthesis. In addition, the fluctuations in oleic acid, Arachidonic acid, alpha-Linolenic acid, docosahexaenoic acid and eicosapentaenoic acid (EPA) were tightly linked to the biosynthesis of unsaturated fatty acids. Notably, EPA, adenosine and anandamide (AEA) were consistently upregulated in both the blank control vs. model group and the combination vs. model group comparisons (Figure 6E).

## 4 Discussion

Ulcerative colitis is a chronic, relapsing inflammatory bowel disease that primarily affects the colon. It triggers the release of pro-inflammatory cytokines in the colon, leading to gut microbiota imbalances and further compromising the intestinal barrier (32, 33). Although pharmaceutical treatments are commonly used in clinical settings, many of these medications come with adverse side effects (30). Currently, nutritional strategies, particularly those targeting the gut microbiota, have emerged as promising adjunctive approaches (8, 34).

Emerging evidence suggests that patients with UC often suffer from impaired VA absorption (35). As an essential nutrient, VA not only supports normal visual function but also plays a crucial role in cell differentiation, organ development, immune defence, and the maintenance of epithelial integrity (36). Most of the non-visual physiological effects of VA are mediated by its active metabolite, RA, which contributes to the maintenance of intestinal epithelial barriers, regulation of gut immunity, and stabilisation of host-microbiota interactions (37, 38). Interestingly, gut microbiota can enhance host retinol bioavailability, thereby improving VA absorption and promoting the hepatic storage of retinyl esters (39). Previous studies have also shown that certain commensal bacteria are capable of autonomously metabolising VA, producing intermediates such as retinol, retinaldehyde, and RA, thus influencing VA metabolism within the intestinal environment (20). Leveraging probiotics with the ability to metabolise VA may provide a novel strategy for UC management by supporting vitamin metabolism, modulating immune responses, reinforcing intestinal barrier integrity, and reshaping gut microbial communities.

In a DSS-induced colitis mouse model, RA supplementation significantly reduced inflammatory cell infiltration in colonic tissues, showing superior therapeutic effects compared to VA alone. Pathological analysis also revealed that the combination of dietary VA and probiotics effectively alleviated crypt distortion and inflammation. Furthermore, probiotic supplementation markedly increased RA levels in the colon, supporting the notion that intestinal VA metabolism can be modulated through microbial interventions.

I-FABP, LBP, and DAO were used as biomarkers to evaluate whether the combination of CCFM1426 and VA contributed to maintaining the intestinal barrier. DAO is an intracellular enzyme that exists almost exclusively in intestinal mucosal cells and is released into the bloodstream when the intestinal barrier is compromised (40). Elevated serum DAO levels indicate damage to the intestinal mucosa (40). LBP is an acute-phase protein synthesised by hepatocytes in response to bacterial infection and endotoxins; its secretion increases with intestinal barrier disruption (41). These markers, therefore, serve as indicators of intestinal permeability and mucosal injury. In this study, RA supplementation significantly reduced serum I-FABP, LBP, and DAO levels in mice with DSS-induced colitis. Notably, treatment with the combination of CCFM1426 and VA also markedly decreased serum concentrations of I-FABP, LBP, and DAO in colitis mice, suggesting a protective effect on the intestinal barrier. These findings align with previous research showing that probiotics help maintain intestinal integrity and that VA metabolites play a key role in repairing mucosal damage. Moreover, our study demonstrated that the combination exerted better efficacy than VA alone, suggesting that probiotics may enhance gut-derived RA production through modulation of the gut microbiota, thereby improving the intestinal barrier.

To further investigate the immunomodulatory and antioxidant roles of VA, RA, and their combination with CCFM1426 in colitis, the expression of key cytokines and antioxidant enzymes was evaluated. In mice with DSS-induced colitis, levels of the pro-inflammatory cytokines TNF-*α*, IL-1β, and IL-6 were significantly elevated, whilst the anti-inflammatory cytokine IL-10 was markedly decreased, indicating an intense inflammatory response and immune dysregulation. These findings are consistent with previous studies that reported excessive release of inflammatory cytokines leading to oxidative stress and epithelial injury in UC (42).

Treatment with VA, RA, or the combination effectively modulated cytokine levels in colitis-affected mice. Notably, the CCFM1426 and VA combination significantly reduced pro-inflammatory TNF-α and IL-6 whilst simultaneously increasing anti-inflammatory IL-10 levels (*p* < 0.05), suggesting that probiotic-enhanced VA metabolism contributed to inflammatory balance restoration. RA alone demonstrated potent efficacy by reducing TNF-α, IL-1β, and IL-6 levels whilst significantly elevating IL-10 expression (*p* < 0.05). These results align with RA’s established role in inhibiting Th17-related inflammation and promoting regulatory T cell responses to maintain immune homeostasis (21, 43). Furthermore, colonic RA levels were drastically reduced in DSS-treated mice compared to controls (*p* < 0.001), whereas both RA supplementation and the combination treatment restored RA levels, with the combination outperforming VA alone. This suggests CCFM1426 enhances the gut microbiota’s VA metabolic capacity, promoting endogenous RA synthesis and facilitating its transport across the intestinal epithelium for local immunomodulatory effects. Concurrently, antioxidant enzyme activities (CAT, SOD, and GSH-PX) were significantly impaired in the model group, indicating ROS accumulation and oxidative stress in inflamed colonic tissues. RA treatment markedly restored these antioxidant enzyme levels (*p* < 0.05), whilst the CCFM1426 and VA combination significantly enhanced CAT and GSH-PX activities (*p* < 0.05), suggesting superior antioxidant capacity, although no statistical significance was observed when compared to VA alone. This highlights the synergistic effect of probiotics and VA in regulating redox homeostasis, likely through increased RA production and reduced oxidative damage. These findings emphasise that probiotics targeting VA metabolism not only modulate inflammatory cytokine networks but also reinforce antioxidant defence systems to protect intestinal integrity.

Gut microbiota homeostasis plays a crucial role in regulating ulcerative colitis (UC), whilst dysbiosis contributes to colitis exacerbation. Our study revealed significantly higher Shannon, Richness and Simpson indices in the model group compared to controls (*p* < 0.0001). *β*-diversity analysis using Bray–Curtis distance demonstrated that the clustering of the model group differed markedly from the CCFM1426 and VA combination groups, indicating significant differences in faecal microbiota compositional structure. The combination treatment effectively restored gut microbiota homeostasis in colitis-affected mice. Several beneficial bacteria were identified in the treatment groups: *Phocaeicola vulgatus*, which metabolises agmatine to regulate intestinal motility and maintain barrier integrity whilst providing neuromodulatory effects (44); *Parabacteroides merdae*, which produces histamine that functions as a regulatory neurotransmitter (44); *Bacteroides congonensis*, which shows increased abundance in inulin-fed mice with potential beneficial effects (45, 46). Furthermore, the combination intervention group exhibited higher relative abundance of *Limosilactobacillus reuteri* and lower abundance of *Ligilactobacillus murinus* compared to the model group. *L. reuteri* is recognised for its probiotic potential, particularly its ability to suppress inflammation by stimulating group 3 innate lymphoid cells (ILC3s) to secrete anti-inflammatory cytokines such as IL-10 and IL-22 (47, 48). Although *L. murinus* has been associated with intestinal barrier repair, excessively elevated levels may reflect incomplete recovery of gut barrier integrity and microbial balance following DSS-induced colitis. Thus, the reduced abundance of *L. murinus* in the combination group may indicate more complete restoration of intestinal homeostasis after DSS withdrawal. Notably, this combination significantly increased colonic retinoic acid levels in colitis-induced mice. Collectively, our results suggest that the VA and CCFM1426 combination alleviates gut dysbiosis by restoring microbial homeostasis through gut microbiota remodelling and enhancing gut-derived VA metabolism.

Alterations in the gut microbiota can significantly influence host metabolic phenotypes and the expression of associated metabolites (49). The study revealed that the combined use of CCFM1426 and VA markedly modulated the faecal metabolite profile in colitis-induced mice and promoted the accumulation of several beneficial metabolites. Differential metabolite analysis demonstrated that these changes were primarily enriched in biosynthesis of unsaturated fatty acids and purine metabolism. Notably, EPA, AEA and adenosine were identified as key differential metabolites, exhibiting significantly elevated levels in all intervention groups except the model group. EPA, an essential polyunsaturated fatty acid, exhibits anti-inflammatory properties that can alleviate chronic inflammatory conditions. EPA supplementation effectively mitigates inflammation in acetic acid-induced rat colitis models by modulating the TGF-*β*/p-EGFR and NF-κB inflammatory pathways, restoring oxidant/antioxidant balance, and enhancing colonic barrier integrity (50). Clinically, the free fatty acid form of EPA has been shown to reduce faecal calprotectin levels and prevent relapse in ulcerative colitis patients (51). Furthermore, emerging evidence indicates that EPA can potently enhance intestinal stem cell-mediated colonic epithelial regeneration through activation of the LSD1-WNT signalling pathway (52). AEA, a pivotal component of the endocannabinoid system, plays a crucial role in gut homeostasis (53) Attenuation of AEA signalling in the intestine significantly exacerbates visceral hypersensitivity (53). Adenosine is considered a pivotal immunomodulator that may control inflammation in IBD (54). Adenosine deaminase inhibition alleviates the elevation of colonic myeloperoxidase and malondialdehyde levels in rats with colitis, demonstrating that adenosine can modulate the immune system and suppress inflammation by reducing pro-inflammatory cytokine biosynthesis and regulating neutrophil function (55). Therefore, CCFM1426 and VA may improve colitis symptoms in mice by remodelling the gut microbiota and regulating metabolic phenotypes, thereby reducing intestinal permeability and exerting anti-inflammatory effects.

## 5 Conclusion

This study demonstrates that combined administration of VA and probiotic CCFM1426 alleviates DSS-induced colitis in mice by reducing inflammation, enhancing intestinal barrier integrity, and restoring gut microbial homeostasis. Notably, the combination elevated colonic retinoic acid levels more effectively than VA alone, suggesting enhanced VA metabolism by CCFM1426. Microbiota analysis revealed increased abundance of beneficial species such as *Bacteroides congonensis* and *Phocaeicola vulgatus*, along with improved *α*- and *β*-diversity. In addition, this combined intervention modulated gut microbiota composition and enriched faecal metabolites, including EPA, adenosine, and AEA, indicating a shift towards a health-promoting intestinal microenvironment. These findings highlight a novel microbiota-targeted nutritional strategy in which probiotics enhance the efficacy of vitamin A through synergistic regulation of retinoid metabolism and microbial homeostasis.

## Data Availability

The original contributions presented in the study are publicly available. This data can be found at: https://www.ncbi.nlm.nih.gov, accession number PRJNA1275596.

## Footnotes

^1^ https://www.metaboanalyst.ca/

^2^ https://www.omicstudio.cn/tool
